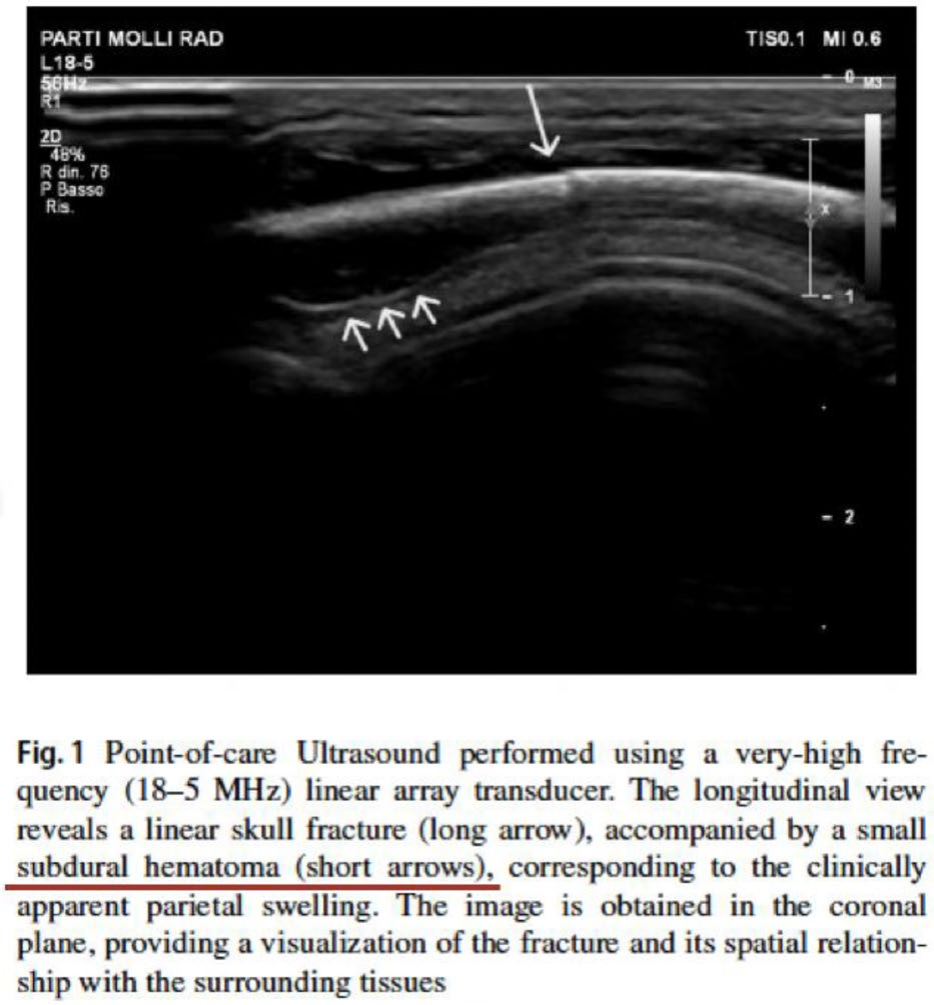# Re: Identifying skull fractures after head trauma in infants with ultrasonography: is that possible?

**DOI:** 10.1007/s40477-025-01014-x

**Published:** 2025-04-14

**Authors:** Salvatore Bonforte

**Affiliations:** Società Italiana di Pediatria, Rome, Italy

Dear Editor:

I am writing to report an ultrasound technique error, in my opinion rather gross, reported in the article in question.

In the caption of Figure 1 of the article (attached below) we read that, under the skull, it is possible to highlight the small subdural hematoma, indicated by the small arrows.

The message could really be misleading especially for young colleagues or for ultrasound beginners.

It is known that echoes cannot cross the bone table, therefore what the authors described in the ultrasound of Figure 1 is not the subdural hematoma that can accompany a skull fracture (detectable only with other imaging techniques, CT or MRI), but is an artefact of the "mirror effect" of the swelling of the soft tissues, linked to the trauma.

I would also like to add that in these cases, in addition to documenting the fracture line, the ultrasound must also detect whether the blood collection due to the trauma is above the aponeurotic galea or between it and the periosteum or below it.

The ultrasound can highlight a subdural collection only through the transfontanellar ultrasound study, but the one described and indicated by the small white arrows in the caption of Figure 1 is the mirror effect of the subgaleal blood collection.